# Sinus bone graft and simultaneous vertical ridge augmentation: case series study

**DOI:** 10.1186/s40902-019-0221-5

**Published:** 2019-09-16

**Authors:** Dong-Woo Kang, Pil-Young Yun, Yong-Hoon Choi, Young-Kyun Kim

**Affiliations:** 1 Department of Oral and Maxillofacial Surgery, Section of Dentistry, Seoul National University Bundang Hospital, 300, Gumi-dong, Bundang-gu, Seongnam-si, Gyeonggi-do 463-707 South Korea; 2 Department of Conservative Dentistry, Section of Dentistry, Seoul National University Bundang Hospital, Seongnam, South Korea; 3 Department of Dentistry & Dental Research Institute, School of Dentistry, Seoul National University, Seoul, South Korea

**Keywords:** Sinus bone graft, Vertical ridge augmentation, Dental implant

## Abstract

**Background:**

This study aims to examine the outcome of simultaneous maxillary sinus lifting, bone grafting, and vertical ridge augmentation through retrospective studies.

**Methods:**

From 2005 to 2010, patients with exhibited severe alveolar bone loss received simultaneous sinus lifting, bone grafting, and vertical ridge augmentations were selected. Fifteen patients who visited in Seoul National University Bundang Hospital were analyzed according to clinical records and radiography. Postoperative complications; success and survival rate of implants; complications of prosthesis; implant stability quotient (ISQ); vertical resorption of grafted bone after 1, 2, and 3 years after surgery; and final observation and marginal bone loss were evaluated.

**Results:**

The average age of the patients was 54.2 years. Among the 33 implants, six failed to survive and succeed, resulting in an 81.8% survival rate and an 81.8% success rate. Postoperative complications were characterized by eight cases of ecchymosis, four cases of exposure of the titanium mesh or membrane, three cases of peri-implantitis, three cases of hematoma, two cases of sinusitis, two cases of fixture fracture, one case of bleeding, one case of numbness, one case of trismus, and one case of fixture loss. Prosthetic complications involved two instances of screw loosening, one case of abutment fracture, and one case of food impaction. Resorption of grafted bone material was 0.23 mm after 1 year, 0.47 mm after 2 years, 0.41 mm after 3 years, and 0.37 mm at the final observation. Loss of marginal bone was 0.12 mm after 1 year, and 0.20 mm at final observation.

**Conclusions:**

When sinus lifting, bone grafting, and vertical ridge augmentation were performed simultaneously, postoperative complications increased, and survival rates were lower. For positive long-term prognosis, it is recommended that a sufficient recovery period be needed before implant placement to ensure good bone formation, and implant placement be delayed.

**Electronic supplementary material:**

The online version of this article (10.1186/s40902-019-0221-5) contains supplementary material, which is available to authorized users.

## Background

After extracting a tooth in the maxilla, the alveolar bone undergoes resorption, and buccopalatal or vertical bone loss results in an edentulous area of the maxilla [1]. Normally in an edentulous area, atrophy of alveolar bone first affects the width of the alveolar ridge and then the vertical aspect of the alveolar ridge [2]. In patients with severe vertical defects in the alveolar bone due to various causes such as tooth loss, periodontal disease, trauma, and surgical resection of tumors, it is difficult to place implants of appropriate axis, depth, and width. In such cases, it is advantageous to reconstruct the alveolar bone through bone grafting and soft tissue surgery and to place the implants in a second surgery. If the amount of alveolar bone is insufficient, various surgeries such as bone grafting, guided bone regeneration (GBR), onlay bone grafting, ridge splitting, ridge expansion, distraction osteogenesis (DO), interpositional bone grafting, and sinus lifting with or without bone grafting have been performed [3, 4].

It is known that a titanium mesh or non-absorbable barrier membrane is effective for providing stability to bone grafting material to effectively increase the vertical height of the alveolar bone [5]. Vertical alveolar ridge augmentation is first performed by GBR using particle-type bone grafting material, while the onlay bone grafting technique requires block bone. It is recommended that bone grafting materials for augmentation of alveolar bone include autogenous bone, autogenous tooth bone graft (autoBT®), allograft, xenograft bone, and alloplast bone, but the best results involve graft material with as much autogenous bone as possible. Block bones can be used for large amounts of bone augmentation. However, block bone graft involves complications such as secondary bone depression in donor sites and nerve damage of the inferior alveolar nerve, mental nerve, or long buccal nerve. In addition, block bone grafts have a disadvantage of a limited amount of collection, and significant bone resorption can occur after bone grafting. It has been reported that approximately 25% of the grafted bone will be resorbed [6].

In some cases, both sinus bone graft and vertical ridge augmentation are necessary due to severe sinus pneumatization and severe alveolar ridge atrophy. In this case, sinus bone graft and vertical ridge augmentation were performed simultaneously, but the surgery had high surgical difficulty and increased risk of failure of both the bone graft and implant [7, 8]. First, primary soft tissue closure is very difficult. If vertical ridge augmentation is performed, soft tissue for wound closure can be deficient. Therefore, completely tension-free primary closure is achieved by performing sufficient undermining or using a local flap, but wound dehiscence can result from postoperative swelling and tension after suturing. If wound dehiscence occurs, risk of grafted bone material loss, postoperative infection, and implant failure will be increased. Next, bone grafts can be successfully established only when the blood flow supply is enough, but it is difficult to secure dual blood supply to both the upper and lower sides due to thin alveolar bone. Therefore, there are issues with delayed healing or insufficient bone graft healing when performed on both sides.

In this study, we analyzed clinical prognosis, effective treatment methods, and research methods by retrospectively analyzing cases of simultaneous sinus bone grafting and vertical ridge augmentation in heavily atrophied molar areas of the maxilla for dental implant placement.

## Materials and methods

This study was conducted under the approval of the Bioethics Review Committee of Seoul National University Bundang Hospital (IRB: B-1811-505-103). From 2005 to 2010, patients who underwent no treatment for a long period of time after loss of teeth or who exhibited severe atrophy of alveolar bone caused by progressive periodontitis were selected as subjects of the study. Patients with insufficient bone mass during implant placement also had to meet the following conditions.

1. Underwent surgery by one surgeon in the Department of Oral and Maxillofacial Surgery, Seoul National University Bundang Hospital
2. Underwent simultaneous vertical ridge augmentation with maxillary sinus bone grafting

There were a total of 15 patients (11 men and four women) with 33 implants placed. The medical records were analyzed retrospectively, and resorption of the grafted bone material in the maxillary sinus, resorption of alveolar bone augmentation, and marginal ridge bone loss were measured using radiographs (periapical radiograph and panorama). The panoramic equipment used in this study were the Orthoceph OC100 CR (Instrumental Imaging, Tuusula, Finland) and RAYSCAN α-OCL (Ray Co., Ltd., Gyeonggi-do, Korea). Periapical radiograph equipment consisted of the RVG6200 (CARESTREAM HEALTH, Inc., Trophy, France) sensor and Heliodent DS (Sirona, Bensheim, Germany).

**Fig. 1:**
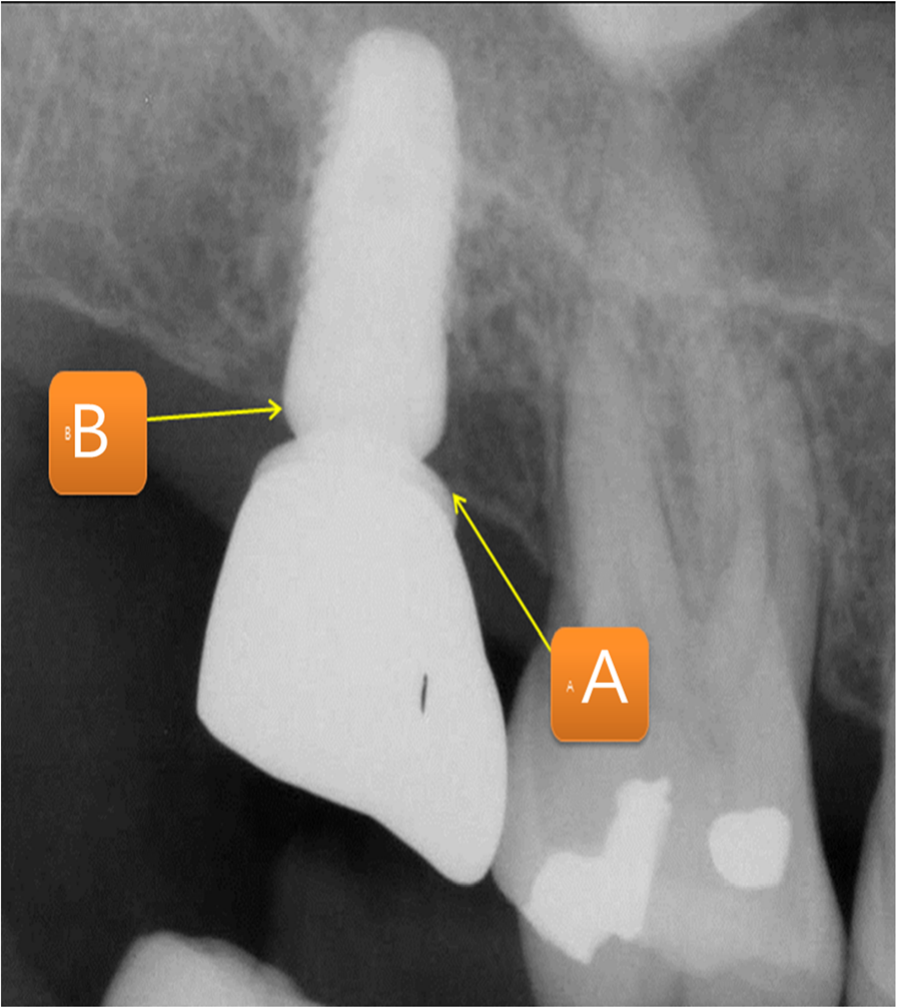
Measurement of marginal bone loss. Measurements of the mean mesial and distal bone which were calculated value obtained as a ratio to the length of the actual implant fixture

**Fig. 2:**
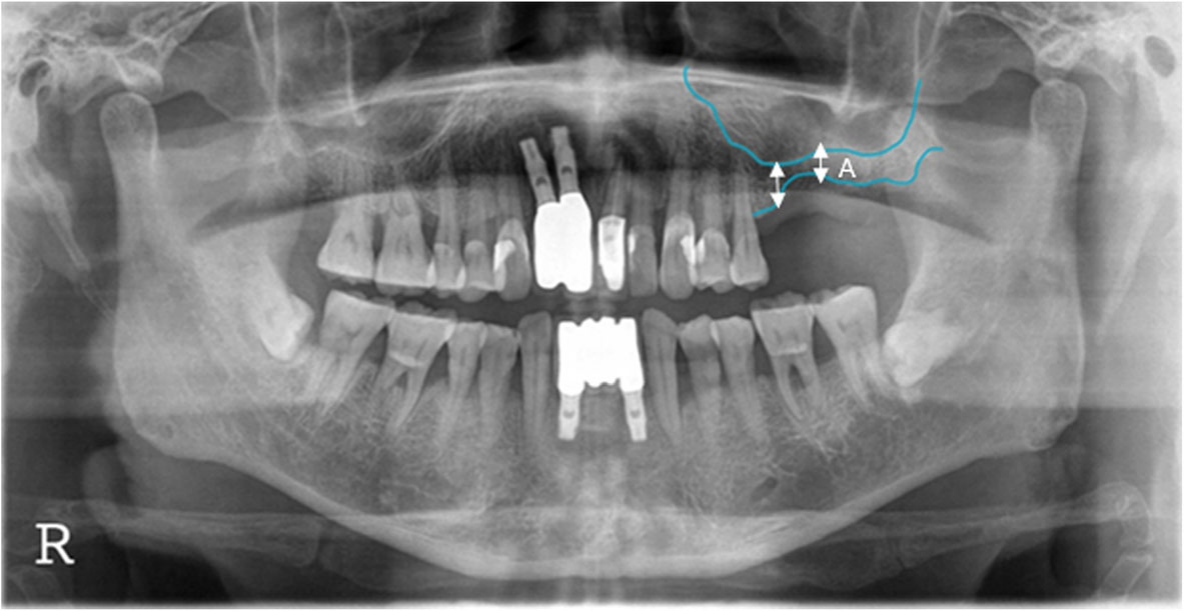
Preoperative panorama radiograph. Diagram for measuring the height of residual alveolar ridge height (A: preoperative residual alveolar ridge height)

**Fig. 3:**
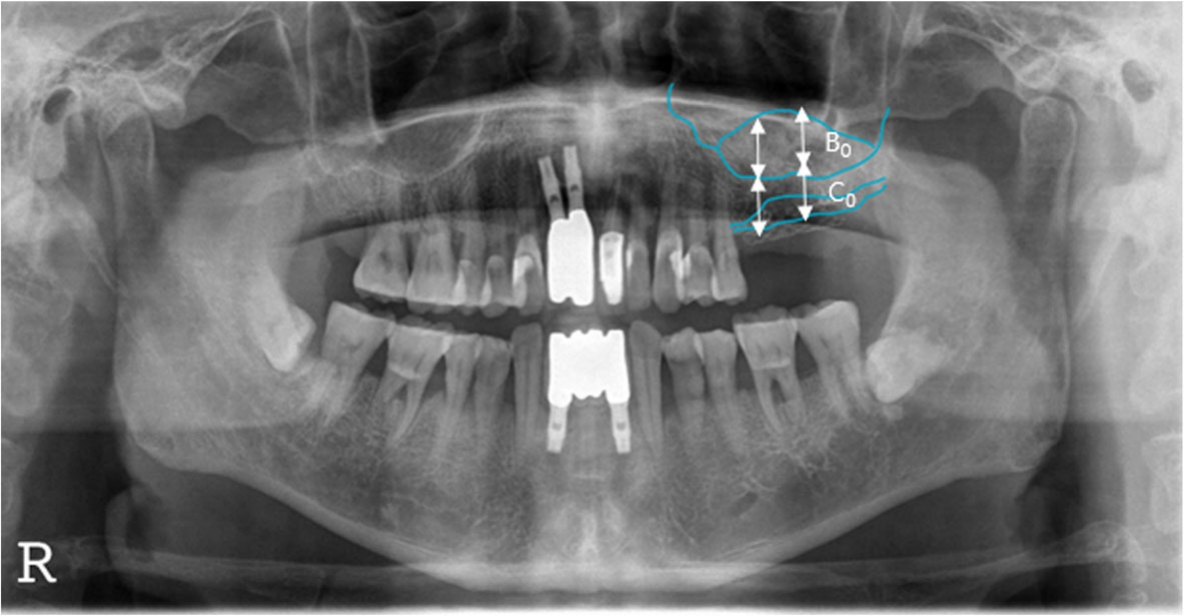
Postoperative panorama radiograph. Diagram for measuring the height of change of sinus bone graft material and vertical ridge bone graft material. (B_0_: sinus bone graft material height, C_0_: vertical alveolar ridge height)

Patients’ age, sex, underlying diseases, locations of implant placement, additional surgeries accompanied by bone grafting, healing periods after bone grafting, implants’ product name, implants’ length and diameter, implant stability quotation (ISQ), bone graft materials, barrier membranes, other additives, complications, prosthesis types, observation period, implant success rate and survival rate, marginal bone loss, resorptions of vertical ridge augmentation at 1 year post-completion of the prosthesis, and final observation were all analyzed. In this study, the success criteria for implants were based on the criteria of Albreksson and Zarb in the 1986 Toronto reference [9]. In the presence of implants in the oral cavity, there should be no clinical mobility, no radiolucent lesion around implants, no gradual loss of bone (less than 0.2 mm per year after 1 year), no infection exhibiting pain or purulent exudate, a 5-year success rate of 85% or more, and a 10-year success rate of 80% or more. On the other hand, the implant survival criteria are defined as stability in the mouth until planned removal [10]. The implant stability quotient (ISQ) was measured with a Smartpeg™ (Ostell AB. Göteborg, Sweden) and an Osstell Mentor^Ⓡ^ (Ostell, Gütberg, Sweden), and primary stability was measured immediately after placement of the implant fixture, while secondary stability was measured at the time of the second surgery in which a healing abutment was connected or impression was performed. Amount of marginal bone resorption was obtained by measuring the height variation from the first thread of the implant to the mesiodistal crestal bone based on the point of the prosthesis through periapical radiography, with measurements of the mean mesial and distal bone which were calculated value obtained as a ratio to the length of the actual implant fixture (Fig. 1). Vertical bone resorption of grafted bone in the maxillary sinus and vertical bone resorption of ridge augmentation were measured and evaluated through panoramic radiography based on the area where the final implant was placed and compared with the final observation point, immediately before and immediately after surgery, and 1 year after prosthesis function. Resorption of the grafted bone material in the maxillary sinus and vertical bone resorption of the alveolar bone augmentation were measured before, immediately after surgery, 12 months after surgery, and at final observation using a radiographic imaging program (PACSPLUS viewer, Medical Standard Co., Ltd., Seoul, Korea) to measure the previous images and anatomical structures. If necessary, each of the following were measured by superimposing the anatomical structures of previous images (Figs. 2 and 3).

1. Height of residual alveolar bone before surgery. (A): Vertical length from the edentulous alveolar crest to the lowest part of the maxillary sinus floor.
2. Height of the grafted bone materials in the maxillary sinus. (B): Vertical length from the lowest part of the maxillary sinus floor to the top of the grafted material in the maxillary sinus that overlaps with the preoperative image or is observed in postoperative images.
3. Height of the alveolar bone after vertical ridge augmentation. (C): Vertical length from the lowest part of the maxillary sinus floor to the highest part of the vertical ridge grafting material that overlaps with the preoperative image or is observed in postoperative images.
4. Height of the vertical ridge augmentation graft. (D): Calculate the values(C − A): Height of the alveolar bone after vertical ridge augmentation. (C): Height of the residual alveolar bone before surgery. (A)
5. Variation of the height of the bone graft in the maxillary sinus: Measurement of the height of the grafted bone materials in the maxillary sinus immediately after surgery (B_0_), the implant prosthesis after 12 months (B_1_), and the final observation point (B_2_) with the calculation of B_0_ − B_1_ and B_0_ − B_2_.
6. Variation of the height of the vertical ridge augmentation material: Measurement of the height of the vertical ridge augmentation graft immediately after surgery (D_0_ = C_0_ − A), implant prosthesis after 12 months (D_1_ = C_1_ − A), and the final observation point (D_2_ = C_2_ − A) with the calculation of D_0_ − D_1_ and D_0_ − D_2_.
7. Calibration and calculation of the magnification (approximately 1.25 times) of the panoramic images from the calculated values.

## Results

**Fig. 4:**
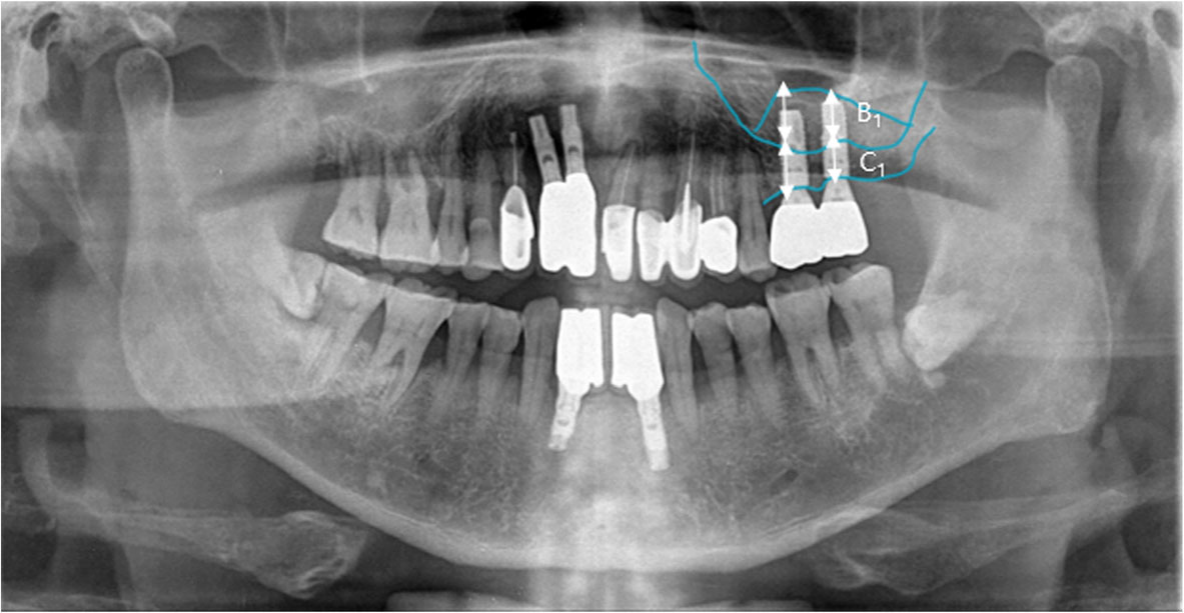
Post-1 year loading panorama radiograph. Diagram for measuring the height of change of sinus bone graft material and vertical ridge bone graft material. (B_1_: sinus bone graft material height, C_1_: vertical alveolar ridge height)

There were a total of 15 patients (11 men and four women) with 33 implants placed. The average age of the patients studied was 54.2 ± 7.4 years, and the average implant loading period was 74.9 ± 40.8 months. In six of the 15 patients, 33 implants failed to survive and succeed. The survival and success rates of the implants were 81.8%. The average primary stability measured during the first implant placement was 61.3 ± 10.5 ISQ, while secondary stability measured during the second surgery or impression appointment averaged 73.5 ± 8.4 ISQ. In nine cases, bone grafting and implant placement were performed simultaneously. In 24 cases, placement of the implant was delayed after bone grafting, for which the average healing period from bone graft to implant placement was 4.3 ± 0.7 months. Marginal bone loss of the calculated mean of the mesial and distal sides excluded from the success criteria, averaging 0.27 ± 0.12 mm 1 year after loading and 0.42 ± 0.21 mm at the time of final observation (Table 1) (Fig. 4).

**Table 1:** Postoperative complications

Complication	Number
Eccymosis	8
Exposure of Ti-mesh or membrane	4
Peri-implantitis	3
Hematoma	3
Maxillary sinusitis	2
Fracture of fixture	2
Bleeding	1
Numbness	1
Trismus	1
Loss of fixture	1

Surgery accompanied by bone grafting was performed with ten pedicled buccal fat pad (PBFP) grafts, three ridge splits, and one free gingival graft (FGG). PBFP grafting was mainly used to close a large mucous membrane perforation of the maxillary sinus when elevating the maxillary sinus. Ridge splitting was performed with bone grafting in cases where the narrow width of the alveolar ridge made it difficult for the implant to be placed. FGG was performed in cases with a very small amount of keratinized gingiva and difficult plaque management. Most of the materials for bone grafting were mixed with autogenous bone graft, autogenous tooth bone graft (autoBT®), allograft, xenograft bone, and alloplast bone. Most of the cases used particle-type bone grafts, while block bones were used in two cases.

Barrier membranes such as a Goretex membrane, collagen membrane, and titanium mesh were used in all the cases except one. For surgery, the tissue adhesive Greenplast Kit® (Green Cross, Gyeonggi-do, Korea) was used in 19 cases in the bone grafting material and the mucous membrane area of the maxillary sinus.

Tissue adhesive was used for stabilization of the resorbable membrane used for sealing a perforated sinus membrane and immobilization of particulate bone graft material. Surgicel® (Ethicon, Somerville, NJ, USA) was used in two cases to close and control bleeding of the perforated maxillary sinus mucosa.

Early complications immediately after surgery comprised eight cases of ecchymosis, four cases of wound dehiscence, three hematomas, one case of bleeding, one case of numbness, and one case of trismus (Table 1). Complications were counted as duplicates that occurred in one implant. Hematoma and ecchymosis were accompanied in one patient, and peri-implantitis occurred first, then several instances of screw loosening, and eventual fracture of the implant fixture in two implants. Vertical resorption of sinus bone graft was 0.23 ± 0.40 mm 1 year after surgery and 0.37 ± 0.61 mm at final observation (Table 2). Resorption of vertical ridge augmentation was 0.12 ± 0.29 mm after 1 year of loading and 0.20 ± 0.37 mm at final observation (Table 3).

**Table 2:** The vertical change of grafted bone material in postoperative follow-up periods (mm)

Postoperative duration	Mean change	Number
1 year	− 0.23 ± 0.40	33
Final	− 0.37 ± 0.61	33

**Table 3:** The vertical change of vertical ridge augmented bone loss in postoperative follow-up periods (mm)

Postoperative duration	Mean change	Number
1 year	− 0.12 ± 0.29	29
Final	− 0.20 ± 0.37	29

Five of the six implants that failed were replaced and continue to function well. One implant was replaced but failed again and is functioning well after the third implant placement procedure. Three implants failed to osseointegrate to the alveolar bone before loading was applied, while three other implants failed after prosthesis function (late failure) (Table 4).

**Table 4:** The vertical change of peri-implant marginal bone loss in postoperative follow-up periods (mm)

Postoperative duration	Mean change	Number
1 year	− 0.27 ± 0.12	29
Final	− 0.42 ± 0.21	29

## Discussion

In cases of severe loss of alveolar bone in the area of maxillary molars, sufficient alveolar bone augmentation is required to ensure successful implant placement and maintenance of implants. According to a 2004 study by Simion et al., vertical bone loss in maxillary molar areas was divided into four categories [11]. Vertical ridge augmentation is considered when vertical bone loss is greater than 3 mm from the cementoenamel junction of adjacent teeth to the crestal bone. If the residual alveolar bone is less than 6 mm in height, sinus elevation is necessary. In 7 years of long-term observation when two bone grafts were performed simultaneously, the bone reaction of implants did not significantly differ from implants that had no grafting.

If only maxillary sinus elevation and sinus bone grafting are performed on vertically atrophied alveolar bone, the length of the prosthesis may be longer, producing a ratio of crown to implant greater than 1:1, increasing the load transferred to the structure of the alveolar bone and implant prostheses [12]. This makes it difficult for the implant to resist occlusal forces and reportedly increases the risk of alveolar bone resorption, fracture of the porcelain of the prosthesis, loosening of screws, etc. [13, 14]. On the other hand, other studies have shown that, even with a subpar ratio of crown to implant, there is no significant clinical difference in implant success [15, 16].

In this study, nine of the 33 implants were simultaneously placed with bone grafts. In 24 cases, implant placement was delayed after initial bone grafting. In cases of delayed implant placement, an average healing period of 4.3 months was allowed after bone grafting. Cowood et al. reported that, if residual alveolar bone is insufficient, bone grafting performed with delayed implant placement after 3–6 months of healing time could increase the success rate [17]. McGrath et al., on the other hand, stated that, if the implant is placed at the same time as the bone graft, the implant minimizes resorption of grafted bone material and reduces alveolar bone loss [18]. In this study, if the initial stability was judged to be sufficient based on residual bone mass and ISQ, the implant was simultaneously placed with bone grafts; the placement of implant was delayed if the residual bone mass was insufficient.

The success of bone grafting is more important than the choice of materials to operate. Exposure to postoperative infections, exposure to wound dehiscence, and increased adherence of bone and grafting materials were important points. Increased mobility of grafted materials or bony segments hinder re-vascularization, resulting in necrotic bone, making it difficult to incorporate with alveolar bone due to survival of only calcified materials [19, 20]. Therefore, surgery of soft tissue is also an important factor in bone grafting, requiring tension-free suturing. In this study, resorbable membranes were used in the lateral sinus opening to reduce the mobility of the bone grafting particles and induce superior adhesion during bone grafting after sinus elevation, and resorbable membranes and tissue adhesives were used to close the perforated sinus mucous membrane. According to Jensen et al., covering the barrier membrane at the lateral sinus opening after bone grafting in the maxillary sinus prevents soft tissue penetration and reduces the mobility of the bone grafting material, resulting in increased success of good bone formation and implants [21].

The material used in maxillary sinus grafting is most ideal when containing autogenous bone. However, the biggest disadvantage of autogenous bone is the limited amount due to few donor sites [22]. There are also reports of greater resorption than with other bone grafting materials and less predictability after surgery [23]. In this study, the block bone of the symphysis of the mandible was collected from two cases, and implant placement was delayed after bone grafting. In one instance, osseointegration failed and resulted in early implant failure. To compensate for the many disadvantages of autogenous bone grafting, autogenous tooth bone graft material (AutoBT®) was used in 11 examples in this study. The bone grafting material is used in powder or putty form by processing the teeth of the patient or their family. Autogenous tooth bone grafting material has excellent osteoinduction and osteoconduction capabilities, has no immunological rejection, and has exhibited excellent clinical results [24, 25].

Complications after surgery included eight cases of ecchymosis, four cases of exposure of the titanium mesh or barrier membrane, three cases of peri-implantitis, three cases of hematoma, and two cases of maxillary sinusitis. Ecchymosis is usually found in patients who have taken drugs that increase bleeding (anti-thrombotic agents), and it is estimated that resuming postoperative medications, even with a temporary suspension of medication, causes severe subcutaneous bleeding, pain, edematous swelling, and hematoma. In this case, short-term use of corticosteroids to prevent postoperative edema may be helpful. It is thought that, if vertical ridge augmentation is performed, the risk of exposure of the barrier or titanium mesh along with postoperative wound dehiscence is high, and resorption increases as the load on the immature bone continues. The use of antibiotics was extended in cases of chronic sinusitis or local infection, and infection control was accompanied by immediate incision, drainage, and daily wound dressing to eliminate complications without any major issues.

The success rate of implants in this study was slightly lower than other studies, at 81.8%, with many complications. Many other studies have shown an average healing period of 5 to 6 months before prosthetic loading of maxillary bone grafts. If vertical ridge augmentation is performed with sinus bone grafting, it is believed that two to three more months of healing time would be advantageous for early stability and success.

In this study, six implants failed to survive, three due to loss of osseointegration before loading. Two of the implants were presumed to exhibit failed osseointegration due to poor initial fixation of approximately 50 ISQ at fixture placement and poor bone quality. The other implant was carefully placed, deliberately removed, and then replaced. The three other failed implants were late failures after prosthesis function, with two of them failing due to repeated parafunction and fracture of the fixtures, while the other implant failed due to repeated peri-implantitis.

In this study, vertical loss of marginal bone was 0.20 ± 0.37 mm at the final observation, with no significant difference compared to studies where implants were placed without bone graft. No significant difference was estimated for the six failed implants that were removed before prosthetic functioning or within 1 year of loading and excluded from the analysis of marginal bone loss. Study by Urban et al. showed no significant difference in resorption of marginal bone around the implants or success rate of implants when comparing cases where only vertical ridge augmentation was performed and cases where vertical ridge augmentation and sinus bone grafting were simultaneously performed [26].

Although vertical resorption of grafted bone materials has shown a gradual increase over time, two-dimensional panoramic radiographs indicate that changes or distortions in the measurement process occur depending on anatomical structure and patients’ position, which will result in a large margin of error and difficulty in assessing reliability. It is believed that, due to the wide variation in the number of cases, it is likely to be difficult to judge reliable results. It is known that resorption of bone grafts occurs continuously for 1 to 3 years after surgery, and that bony changes occur at a minimum level after that [27, 28]. In the future, prospective studies with computed tomography (CT) images analyzing both the type and height of grafted bone materials and changes in the volume of the three-dimensional material will be required.

Also, bone grafts were done with a mixture of autogenous bone, xeno-grafts’ materials, autogenous tooth bone grafts (autoBT®; powder and block), and auto-block bone graft. The marginal bone loss may cause differences in the types of bone grafts materials, but the comparison of the bone grafts by type is difficult due to very small sample size on each methods. For the more accurate assessment and predictive treatment, randomized comparison studies of large sample size, and precise diagnosis will be required according to the condition of the maxillary sinus and the alveolar bone.

## Conclusion

In this study, if maxillary sinus bone grafting and vertical ridge augmentation were performed simultaneously in severely atrophied maxillary molar areas, postoperative complications tended to be high with low implant success and survival rate. Delayed implant placement is thought to result in good prognosis by allowing sufficient healing of 8 months to 12 months for good bone formation after bone grafting.

## Additional file

Additional file 1: Case form and result of data. (XLSX 68 kb)

## Data Availability

The dataset supporting the conclusions of this article is included within the article and Additional file 1.

## Abbreviations

CT: Computed tomography
DO: Distraction osteogenesis
FGG: Free gingival graft
GBR: Guided bone regeneration
ISQ: Implant stability quotient
PBFP: Pedicled buccal fat pad
